# The Iranian version of theory-based intention for cesarean section (IR-TBICS) scale: development and first evaluation

**DOI:** 10.1186/s12884-020-03498-3

**Published:** 2021-01-05

**Authors:** Seyed Abolhassan Naghibi, Maryam Khazaee-Pool, Mahmood Moosazadeh

**Affiliations:** 1 Department of Public Health, School of Health, Mazandaran University of Medical Sciences, Sari, Iran; 2 Health Sciences Research Center, Addiction Research Institutes, Mazandaran University of Medical Sciences, Sari, Iran

**Keywords:** Cesarean section, Intention, Scale development, Psychometrics, Pregnant women

## Abstract

**Background:**

The rate at which mothers experience a cesarean section in the absence of medical signs is growing worldwide. Women’s beliefs and intentions play an essential role in the request or choice of a delivery method. At present, there is no comprehensive, validated scale for assessing pregnant women’s beliefs about cesarean section in the Iranian population. This study was performed to develop and assess the validity and reliability of the intention-based cesarean section scale using the theory of reasoned action (TRA) constructs as a theoretical framework for measuring intention toward the selection of a delivery method.

**Methods:**

In this cross-sectional validation study, 480 pregnant women were recruited from Sari, in northern Iran, through a multistage random sampling approach. Content validity was examined using the content validity index (CVI) and content validity ratio (CVR). Furthermore, both exploratory factor analyses (EFA) and confirmatory factor analyses (CFA) were applied to assess the construct validity of the developed scale. Reliability was measured by internal consistency and the intraclass correlation coefficient (ICC). Quality criteria for floor and ceiling effects were derived from existing guidelines and consensus within our research group.

**Results:**

The results obtained from the factor analysis showed that the data were fit to the model (χ2 = 2298.389, *P* < 0.001). The TRA comprised 24 items assessing five domains, which described 62.46% of the common variance. The CFA showed a model with suitable fitness for the data. Cronbach’s alpha coefficient for the domains of the scale ranged from 0.609 to 0.843, and the ICC value ranged from 0.71 to 0.84, which is within the satisfactory range. The IR-TBICS scale had no floor or ceiling effect on the total score or any of the dimensions.

**Conclusions:**

The belief-based cesarean section scale appears to be a reliable instrument. It is considered suitable and can be applied in other research in Iran.

## Background

One of the most common significant surgeries around the world is cesarean section, the prevalence of which is increasing worldwide. This increase has caused much concern [1–3]. According to the World Health Organization (WHO), a suitable rate of cesarean section is 10–15% [4], which is related to the lowest rate of maternal problems. However, based on the results of various studies, the rates of cesarean section are very high in Iran [5–7]. This rate has been reported as 26–60%, although some private clinics have reported up to 87% [7]. Cesarean delivery on maternal request (CDMR) is responsible for some of the rise in the overall rate. In developed countries, conservative estimates of CDMR range from 4–18% of all cesarean deliveries [8]. Though the rate of CDMR in Iran remains uncertain due to poor evidence, the CDMR rate is high [9], varying from 11.2–22% in various studies [10, 11].

Many studies have examined the reasons for women’s desire for cesarean section. The most common reason in high-income countries is fear of childbirth for various reasons, such as having a traumatic birth experience. [12, 13]. Reasons for Iranian women to have a cesarean section are mentioned, including a lack of awareness and misrepresentation about natural childbirth [14, 15], the need to plan for the delivery date, fear, and the pain of natural childbirth in a prior delivery experience [16]. Furthermore, a part of this rise is due to the changed attitudes of people toward delivery approaches [17]. Other studies have shown that sociocultural, religious, and economic customs [18, 19], perceived behavioral control, emotional causes, misconceptions, and incorrect subjective norms in Iranian mothers were the main elements in their choice of delivery type [20]. The control of behavior in planning is an important factor to reduce the gap between intention and behavior when encountering various conditions. This high increase in cesarean section indicates a medical problem in Iran and requires the attention of health policy makers to pursue programs to reduce the number of unnecessary cesarean Sect. [21].

It appears that the high rate of cesarean delivery in Iran is a complex phenomenon. Thus, educational interventions to reduce the rate of cesarean delivery, improve the quality of routine vaginal delivery services, and change mothers’ outlooks regarding the mode of delivery are essential [22]. Consequently, an instrument carefully assessing factors that affect the choice of delivery methods by mothers is required. Fear, attitude, perceived behavioral control, subjective norms, and behavioral intention are among the most common reasons to select a cesarean Sect. [23–25]. It has been confirmed that negative attitudes are the leading causes to select any particular means of delivery [23]. Other studies have also confirmed that doctors, midwives, and relatives’ thoughts, as well as concepts such as attitude, perceived behavioral control, subjective norms, and behavioral intention, are the key constructs of the theory of reasoned action (TRA).

TRA comprises theoretical constructs such as attitude (beliefs about the behavior’ outcome and evaluations of expected outcomes) and subjective norms (normative beliefs and motivation to comply) [26]. Thus, TRA can be a suitable theory to design interventional programs. As such, a valid and reliable questionnaire is required to extract personal intention. Various studies have been conducted about cesarean sections in Iran [9, 17, 21, 27, 28], but in these studies, researcher-made questionnaires were used, and there remains a lack of suitably validated instruments to measure women’s intentions regarding their selection of delivery method.

Given the lack of a valid scale in Iran and other countries to measure factors predicting choice of delivery method and considering that the choice of delivery method is rooted in sociocultural background, the current study was aimed at the development and psychometric assessment of a questionnaire based on TRA to better understand Iranian pregnant women’s intention toward cesarean section. Such scales could support the identification of the viewpoints of health experts and policymakers and, in turn, help in developing extended interventional plans for regulating the rate of the cesarean birth procedure. It is hoped this might aid in filling in the gaps and might contribute to the current literature on the topic.

## Methods

### Participants and procedures

This cross-sectional validation study was performed in the city of Sari, the capital of Mazandaran province, northern Iran, from February to June 2017. In the present study, the sample size was estimated based on the number of items in the scale, multiplying by 10 (24 × 10 = 240). The most commonly used minimum sample size estimation method in structural equation modeling (SEM) is the *10-times rule* procedure, which makes on the hypothesis that the sample size should be higher than 10 times the maximum number of internal or external model links implying at any latent variable in the model. The 10-times rule was preferred due to its simplicity of use [29, 30]. In all, 480 pregnant women participated in exploratory factor analysis (EFA) (240 pregnant women) and confirmatory factor analysis (CFA) (240 pregnant women). Data for this study came from pregnant women who attended a Baghban specialist clinic, public health care centers, and private gynecological clinics in Sari. Women were chosen using a multistage random sampling method. The first step of the sampling method was aimed at selecting samples from all regions. To this end, a list of public health care centers and gynecological clinics was provided. Subsequently, in proportion to the number of target groups in each of the public and private service centers, the number of samples required was consecutively entered into the study via a simple sampling method.

The inclusion criteria were to be a pregnant woman with gestational age from the seventh to the ninth month and to be interested in participating in the study. Exclusion criteria were a lack of willingness, having a mental illness, or having a specific physical condition such as complete placenta Previa, which was an absolute indication for cesarean delivery with no option for a vaginal birth, making it impossible to participate and complete the questionnaire. The demographic characteristics of the women included age, level of education, and employment status. Data collection approaches were based on nameless scales that were completed by an expert interviewer for protecting the privacy of women and the confidentiality of the data. The interviewer received guidelines for similarly completing the scales after attending a training session.

### Scale development process

This study was performed to develop an instrument to measure the intention of pregnant women to choose the cesarean section delivery method. The scale was developed across several stages. During the first stage, the content domain of the construct was specified. In this stage, interviews were conducted with the experts (gynecologists and midwives) and pregnant women, and a review of the literature relating to the TRA [31–35] was performed to develop an item pool and content domain. The main dependent variable in the present analysis was the cesarean delivery method. In addition, the independent variables included five factors, organized into a logical framework, as follows: (a) behavioral beliefs; (b) evaluation of behavioral outcomes; (c) motivation to comply; (d) normative beliefs; and (e) behavioral intention. The item pool contained 39 items at this point. The principal researcher and other team members then read the items and removed extraneous ones. The first draft of the instrument comprised 27 items. In the second stage, the psychometric properties of the Iranian version of the Theory-Based Intention to Cesarean Section (IR-TBICS) scale were examined to assess its validity and reliability.

#### Content validity

Content validation requires a wide-ranging review by a panel of experts to determine whether the scale items sufficiently address the subject they aim to assess. It is a crucial phase for developing a tool and a method for linking abstract notions with tangible and measurable indices. The expert panel comprised 10 specialists in health education and promotion, gynecologists, and experts familiar with scale making. Qualitative content validity was assessed in terms of the wording, scaling, grammar, and item allocation indices [36]. All items were tested, and the expert panel’s suggestions were added to the scale. We applied the content validity index (CVI) and content validity ratio (CVR) to reach the quantitative content validity of the new scale. To measure CVR, the expert panel was questioned to evaluate each item through a 3-point Likert scale, where 1 = essential, 2 = useful but not essential, and 3 = unessential. The CVR for each item was measured by means of the following formula: CVR = [Ne - (N/2)] ÷ (N/2) (Ne is the number of panelists indicating “essential” for each particular item and N is the total number of the professional panel). The numeric value of CVR is documented by the Lawshe table. Based on Lawshe’s table [37], items with a CVR score of 0.62 or above were selected [36]. For the CVI, consistent with Waltz and Bausell [38], the same panel was questioned to assess the items based on a 4-point Likert scale on “relevancy,” “clarity,” and “simplicity.” The number of those rating the item as relevant or clear (rating 3 or 4) was allocated by the number of a content expert panel. A CVI score of 0.79 or above was applied acceptable [37, 39].

#### Face validity

Face validity is a calculation of laywomen (pregnant women) in understanding and knowing an instrument. In this step, both quantitative and qualitative methods were used. For the quantitative step, ten pregnant women were questioned to assess the instrument and the degree of importance of each item on a 5-point Likert scale to evaluate the “Item Impact Score” (Impact Score = Frequency (%) × Importance). An impact score equal to 1.5 or more was considered acceptable, as declared [40]. For the qualitative step, the same pregnant women were questioned about the ‘relevancy,’ ‘ambiguity,’ and ‘difficulty’ of each item, and some minor modifications were performed on the primary instrument.

#### A preliminary version of the instrument

Reflecting the above methods, a preliminary version of the instrument containing 24 items was created for the next phases (construct validity and reliability of the IR-TBICS scale).

### Statistical analysis

#### Construct validity

The construct validity of the IR-TBICS scale was assessed using both exploratory (EFA) and confirmatory factor analyses (CFA).

#### Main study and data collection

A cross-sectional study was planned to assess the psychometric properties of the IR-TBICS scale. A consecutive sample of pregnant women was recruited from the Baghban specialist clinic, public health care centers, and private clinics of gynecologists affiliated with Mazandaran University of Medical Sciences.

##### a) Exploratory factor analysis (EFA)

A sample of 240 pregnant women completed the IR-TBICS scale, and its factor structure was extracted by principal component analysis with varimax rotation. Bartlett’s test of sphericity and the Kaiser-Meyer-Olkin (KMO) test were applied to assess the suitability of the sample for the factor analysis. Eigenvalues above one and scree plot were conducted to identify the number of factors. Factor loadings equal to or greater than 0.4 were considered appropriate [41].

##### b) Confirmatory factor analysis (CFA)

A separate sample of 240 pregnant women completed the IR-TBICS scale, and factor analysis was conducted to measure the model fitness. As suggested, several fit indices counting relative chi-square (χ2/df), goodness of fit index, normed fit index, non-normal fit index, standardized root mean square residual, comparative fit index, and root mean square error of approximation were accompanied [42, 43]. Relative chi-square is the ratio of the chi-square to degrees of freedom, and its suggested reference value is less than three for accepting the fitness of the model. The values for GFI, CFI, NFI, and NNFI could range between 0 and 1; values closer to 1 reveal data fitness [44, 45]. An RMSEA ranging from 0.08 to 0.10 displays an average fit; less than 0.08 identifies a good fit [43]. The satisfactory value for SRMR is below 0.10; values under 0.08 display satisfactory fit, and values less than 0.05 show good fit [46].

### Reliability

Cronbach’s α coefficient assessed the internal consistency of the IR-TBICS scale. A Cronbach’s α coefficient equal to 0.7 or more was identified as acceptable [47]. Floor and ceiling effects were determined as present if more than 15% of the responders attained the lowest or highest possible total score on the IR-TBICS scale [43]. Furthermore, a subsample of pregnant women (*n* = 25) completed the IR-TBICS scale twice with a 2-week interval to test the stability of the IR-TBICS scale by computing the intraclass correlation coefficient (ICC); an ICC of 0.4 or more was deemed acceptable [48]. All statistical analyses, except CFA, were done using SPSS v22.0 [49]. The CFA was done using the AMOS software v22.0 for Windows [50].

### Scoring

In the final version of the IR-TBICS, a minimum of three and a maximum of seven items were generated for each construct. In the present study, behavioral beliefs and outcome evaluation toward cesarean section were measured with seven and five items, respectively. The items were rated on a 5-point scale ranging from 1 (strongly disagree) to 5 (strongly agree). Higher scores indicate a more positive attitude toward cesarean section. Normative beliefs were assessed concerning other important factors. In the present study, normative beliefs toward cesarean section were measured with six items. The items were rated on a 5-point scale ranging from 1 (strongly disagree) to 5 (strongly agree). In the present study, the motivation to comply with cesarean section was measured with three items. The items were rated on a 5-point scale ranging from 1 (strongly disagree) to 5 (strongly agree). Higher scores indicate more subjective norms persuasive to cesarean section. The intention was assessed using three items. The items were rated on a 5-point scale ranging from 1 (very unlikely) to 5 (very likely). Higher scores indicate more intention to have a cesarean section.

### Ethics

The ethics committee of Mazandaran University of Medical Sciences approved the study. All pregnant participants gave their written informed consent.

## Results

### Sociodemographic characteristics

Overall, 480 pregnant women participated in the study. The age of respondents ranged from 14 to 41 years, with a mean age of 27.72 years (SD = 5.74). Regarding educational level, 11.87% of respondents attained the primary level, 33.33% attained the secondary level, and 54.8% attained the higher level of education. Of the total, 23.9% of the participants were housewives, and the majority of the women (76.05%) were employed. The characteristics of the women are reported in Table 1.

**Table 1:** Characteristics of the study sample

	EFA sample (*n* = 240)	CFA sample (*n* = 240)
	**Number (%)**	**Number (%)**
**Age (years)**		
< 20	12 (5)	10 (4.2)
20–29	132 (55)	136 (56.7)
> 29	96 (40)	94 (39.1)
Mean (SD)	27.72 (5.74)	27.84 (4.77)
Range	14–41	14–41
**Level of education**		
Primary	30 (12.5)	27 (11.2)
Secondary	84 (35)	76 (31.7)
Higher	126 (52.5)	137 (57.1)
**Employment status**		
Housewife	69 (28.8)	46 (19.17)
Employed	171 (71.2)	194 (80.83)

### Feasibility

The results showed no ceiling effect or floor effect for the Iranian version of the belief-based cesarean section scale.

### Content validity

In the quantitative content validity assessment of the IR-TBICS scale, items with a CVR and a CVI less than 0.62 and 0.80, respectively, were removed. Three items were deleted, resulting in a 24-item pool. The expert panel also revised the IR-TBICS scale with regard to grammar, wording, and item allocation. The results of the quantitative content validity assessment indicated that the mean scores for the CVI and CVR were 0.87 and 0.83, respectively.

### Face validity

In the qualitative face validity assessment, women stated small variations in the wording of some items for more description. The result of quantitative face validity indicated that the affect score was equal to or greater than 1.5 for all items of the IR-TBICS scale. None of the items were deleted, and the first draft of the IR-TBICS scale, containing 24 items, was developed for the next phase of psychometric evaluation. In other words, the participants showed that they experienced no trouble reading and understanding the 24 items.

### EFA

**Fig. 1:**
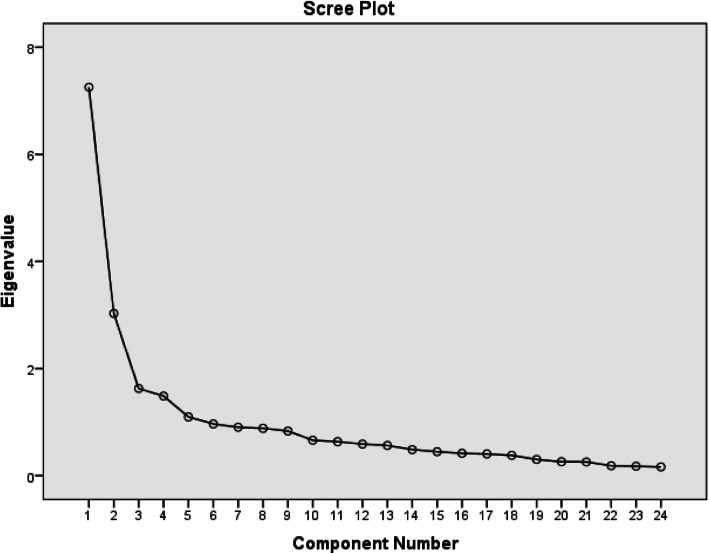
Scree plot for determining factors of the designed instrument

The results of the EFA are presented in Table 2; Fig. 1. The KMO and Bartlett’s test revealed that the data were suitable for factor analysis (KMO index = 0.830, χ2 = 2298.389, *P* < 0.001). Principal component analysis with varimax rotation recognized five factors with eigenvalues greater than 1 and factor loading equal to or more than 0.50, accounting for 62.46% of the variance observed. The factor loadings were as follows: (a) Factor 1 (outcome evaluations) included 7 items (items 7–13); (b) Factor 2 (behavioral beliefs) included 6 items (items 1–6); (c) Factor 3 (injunctive normative beliefs) included 5 items (items 14–18); (d) Factor 4 (behavioral intention) included 3 items (items 22–24); and (e) Factor 5 (motivation to comply) included 3 items (items 19–21).

**Table 2:** Exploratory factory analysis of the IR-TBICS scale (*n* = 240)

Item	Factor1	Factor2	Factor3	Factor4	Factor5
Q8. Delivering my child by the planned cesarean section will aid in creating a healthy relationship between my spouse and me.	.**775**	0.113	− 0.067	0.028	0.053
Q9. Caesarean section is generally easier than vaginal birth method.	.**716**	0.102	0.001	0.133	0.002
Q7. A child born by cesarean section is more intelligent than a child born from vaginal birth.	.**684**	0.096	0.021	0.255	0.099
Q13. Delivering my child by the vaginal birth method can change my bodily form.	.**679**	0.070	0.215	0.159	0.250
Q10. Caesarean section delivery does not have a negative effect on postpartum sexual relationships.	.**548**	0.275	0.165	− 0.393	0.190
Q11. Delivering my child by the planned cesarean section is convenient for me.	.**541**	0.042	0.446	− 0.019	− 0.009
Q12. A planned cesarean section provide relief so I can bond more with my child.	.**539**	0.365	− 0.047	0.053	− 0.183
Q3. In my opinion, a woman with problems like pelvic stenosis should have a caesarean section.	− 0.020	.**735**	0.387	− 0.172	0.138
Q1. In all situations, doing cesarean section is more appropriate than the vaginal birth method.	0.103	.**716**	0.013	0.166	0.123
Q5. Delivering my children by the planned cesarean section is a significant experience for me.	0.300	.**688**	− 0.098	0.018	0.198
Q6. I do not think we have had any serious complications after childbirth by cesarean section.	0.201	.**672**	0.362	− 0.142	0.209
Q2. In my opinion, the problems of vaginal birth method are greater than cesarean section.	0.223	.**630**	0.529	0.094	0.057
Q4. It is important to me that I deliver my children by the planned cesarean section.	0.301	.**559**	0.489	0.014	0.012
Q18. To my spouse, delivering my child at a specific time of day and at a specific time of the year can impact my child’s success in life.	− 0.021	0.091	.**734**	− 0.063	0.157
Q15. My spouse believes that the planned cesarean section is unsafe for me.	0.138	− 0.015	.**656**	− 0.184	0.234
Q17. Delivering my child by the planned cesarean section is an important experience for my spouse.	− 0.119	0.196	.**556**	− 0.024	0.351
Q14. My spouse believes that the planned cesarean section is risky for my child.	− 0.015	0.346	.**534**	0.044	0.226
Q16. My family believe that the planned cesarean section is hazardous for my child.	0.448	0.393	.**529**	− 0.008	0.028
Q20. I believe that it is important to my family that I deliver my child by the planned cesarean section.	0.184	0.040	− 0.056	.**846**	− 0.041
Q19. I believe that it is important to my spouse that I deliver my child by the planned cesarean section.	0.261	0.082	− 0.027	.**814**	− 0.029
Q21. I believe that it is preference of doctor that I deliver my child by the planned cesarean section.	0.447	0.383	0.266	.**458**	0.070
Q22. I intend to have my childbirth by cesarean section method.	0.119	0.234	0.102	− 0.077	.**780**
Q24. I plan to deliver my child using the planned cesarean section method.	− 0.015	0.193	0.289	− 0.004	.**732**
Q23. I would like to deliver my child using the planned cesarean section method.	0.176	0.004	0.360	− 0.044	.**555**

Note. Figures in bold are related to factor loadings equal to or greater than 0.40

### CFA

**Fig. 2:**
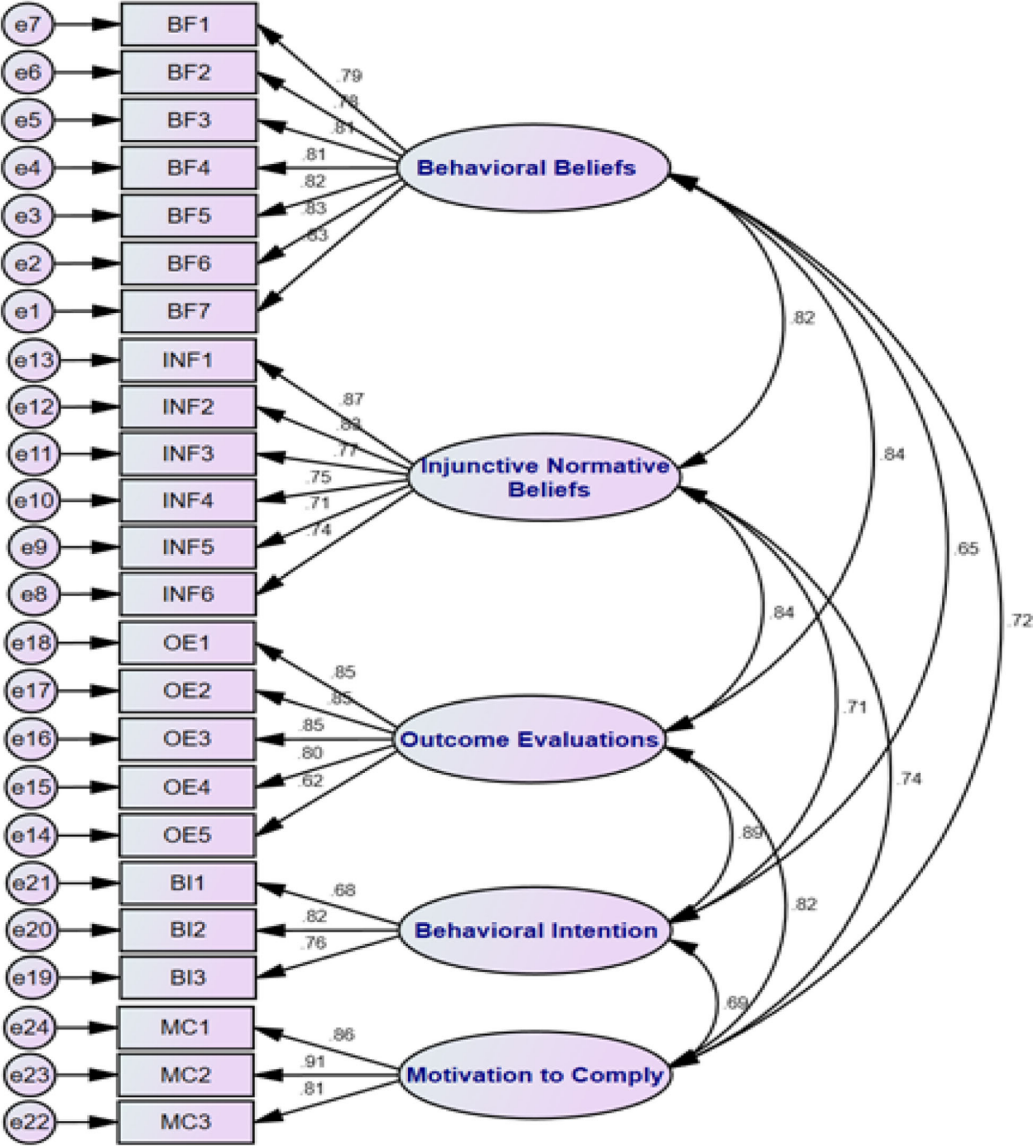
A five-factor model for the scale gained from confirmatory factory analysis (*n* = 240)

The findings of the CFA of the general model with 24 items in five subscales indicated that the model was accepted in its present form (the relative chi-square (x2/df) = 2.606 < 3, *P* < 0.001; RMSEA = 0.077 > 0.08, (95% CI = 0.062–0.078); CFI = 0.931 > 0.9; IFI = 0.914 > 0.9; TLI = 0.892 > 0.8; GFI = 0.913 > 0.9; AGFI = 0.905). Thus, the CFA displays the suitability of the model and the appropriate fit of its structural model for the study samples (Fig. 2).

### Reliability

The reliability of the IR-TBICS scale was assessed using internal consistency. The Cronbach’s α coefficient for the dimensions ranged from 0.609 to 0.843. In addition, the ICC for the theory-based cesarean section beliefs instrument dimensions was assessed, which ranged from 0.71 to 0.84 (acceptable). This supports the stability of the IR-TBICS scale. Internal consistency of behavior comprised one item; therefore, internal consistency reliability was not assessed. Ceiling and floor effects should be less than 15% to comprise all criteria and show the variations during the time; in the current study, no ceiling or floor effects were detected in the total score or any dimensions of the IR-TBICS. The Cronbach’s α and ICC of the theory-based cesarean section beliefs instrument dimensions are presented in Table 3.

**Table 3:** Measures of internal consistency and stability of the IR-TBICS scale

Factor	The name of factor	Number of items	Cronbach alpha (*n* = 240)	ICC (*n* = 25)
**1**	Outcome evaluations	7 items (7–13)	0.74	0.81
**2**	Behavioral beliefs	6 items (1–6)	0.80	0.79
**3**	Injunctive normative beliefs	5 items (14–18)	0.84	0.84
**4**	Behavioral intention	3 items (22–24)	0.61	0.71
**5**	Motivation to comply	3 items (19–21)	0.64	0.78
**Total**		24 items	0.88	0.79

## Discussion

Generally, the theory of planned behavior (TBI) affords a valuable theoretical framework for dealing with the complications of human social behavior [51]. The evaluation of theoretical structures is one of the most problematic and essential sectors in the study of theory-based health education and promotion. The evaluation of TRA structures is best reached by two approaches: a direct technique in which, for instance, the typical attitude of people is assessed regarding certain behaviors, and an indirect (belief-based) technique in which the particular behavioral beliefs and their outcomes are assessed [51, 52]. Indirect assessment of the TRA constructs concentrated on the cognitive structures of TRA. TRA assumes that people can have many opinions about any particular behavior. TRA emphasizes two types of beliefs: behavioral and normative [51]. By knowing cognitive–behavioral beliefs, factors influencing the encouragement to do determined behaviors are recognized and impacted in the intervention research. In the current project, the TRA structures were assessed using a complicated procedure.

The content of the IR-TBICS scale items was first established based on interviews with the experts and pregnant women and a review of the literature to ensure that this instrument covered all theoretical concepts linked to the intention of cesarean section. The results of the analysis due to the KMO index show adequate sample size and satisfactory factor analysis. After EFA, a five-domain scale that was extracted accounted for 62.46% of the variance, and the maximum expressed variations were linked to the behavioral beliefs as a first domain. Ghazanfari (2010) [53] also indicated that the theory of planned behavior (TPB) described 62% of the variance of physical activity, and attitude explained the maximum amount of variance. A CFA disclosed that the fit of the data was satisfactory. As such, the final IR-TBICS scale contained 24 items, with seven items indicating outcome evaluations, six items representing behavioral beliefs, five items representing injunctive normative beliefs, three items representing behavioral intention, and three items representing motivation to comply with cesarean section.

Reliability is discussed with regard to the consistency and stability of the domains of a scale that represent its evaluation accuracy [54]. The findings of Cronbach’s α coefficients between 0.609 and 0.843 for all domains indicated that the IR-TBICS scale had satisfactory reliability. Therefore, we believe the IR-TBICS scale represents a new scale for understanding the intention to choose the method of cesarean section delivery. Even though two domains (motivation to comply and behavioral intention) had a lower level of Cronbach’s α (under 0.70), other domains had higher and acceptable levels of the Cronbach’s α coefficient. However, no significant increase in the Cronbach’s α coefficient was found after deleting any items. The results of the study performed by Bordewich in 2005 [55] indicated that the internal consistency of the TPB was between 0.52 and 0.89. The internal consistency of the TPB domains was also reported to range from 0.54 to 0.82 [53]. In this study, there was no ceiling or floor effect in the total score for any dimensions of the IR-TBICS scale, which shows good content validity of the IR-TBICS scale in pregnant women. The low value of the Cronbach’s α coefficient in some domains may be a result of the low number of items in the domains on how to develop the scale. It is essential that in the current study, the domains of motivation to comply and behavioral intention had three items, although Francis highlighted the existence of at least three items for each factor in the development guide of the TPB scale [52]. Moreover, Ajzen believed that matching items with prior research when developing the scale would result in an instrument with relatively low reliability, which may underestimate the correlation between domains of the theory [51]. It appears that adding the items for some factor can raise the scale reliability; therefore, considering this point, further research has been proposed.

Additionally, the ICC score revealed appropriate stability for the IR-TBICS scale, as it was measured by 25 pregnant women at a 2-week interval (0.79). As such, we believe that this newly developed scale may be particularly valuable for health care groups to know and plan procedures that are useful and targeted to specific conditions. The inclusion of five domains in the IR-TBICS scale further lets professionals identify domains in which a person can be improved.

Although the current study has several strengths, it also has some limitations. First, the present study was done among a sample of pregnant women from the city of Sari (northern Iran) to express their beliefs and intentions about the method of cesarean section delivery. Given this, we cannot be sure that our conclusions can be generalized to pregnant women from other geographic backgrounds. Consequently, further research may be needed to support the applicability of the belief-based cesarean section delivery scale as a fully confirmed applied and useful measure for women of Iranian background. On the other hand, it would be better to investigate doctors’ beliefs and intentions when choosing a delivery method for a woman. There is evidence of doctors’ fear of childbirth and preference for cesarean Sect. [56]. Second, the Cronbach’s α coefficients of some factors were not satisfactory. Future studies are needed to overcome these problems.

In summary, one of the objectives of the century is to reduce unnecessary cesarean Sect. [57]. To do so, we developed the IR-TBICS scale, which was revealed to have acceptable psychometric properties. The IR-TBICS scale assesses the beliefs and intention of choosing cesarean section delivery that help to promote pregnant women’s health.

## Conclusions

Generally, the IR-TBICS scale indicated good construct validity, and the majority of domains indicated high internal consistency reliability; therefore, the results of the present study suggest that the theory-based cesarean section delivery beliefs scale is a valid and reliable questionnaire for measuring the beliefs of pregnant women. Furthermore, further studies are suggested to determine the strengths and weaknesses of the IR-TBICS scale when it is applied to other backgrounds.

## Data Availability

The datasets produced and analyzed throughout the present study are not publicly accessible due to the need to protect the participants’ anonymity but are accessible from the corresponding author on reasonable demand.

## Appendix

### Behavioral beliefs

1. In all situations, doing a cesarean section is more appropriate than the vaginal birth method.
2. In my opinion, the problems of the vaginal birth method are more significant than a cesarean section.
3. In my opinion, a woman with problems like pelvic stenosis should have a cesarean section.
4. It is important to me that I deliver my children by the planned cesarean section.
5. Delivering my children by the planned cesarean section is a significant experience for me
6. I do not think we have had any severe complications after childbirth by cesarean section.

### Outcome evaluations

7. A child born by a cesarean section is more intelligent than a child born from a vaginal birth.
8. Delivering my child by the planned cesarean section will aid in creating a healthy relationship between my spouse and me.
9. Cesarean section is generally more comfortable than a vaginal birth method.
10. Cesarean section delivery does not hurt postpartum sexual relationships.
11. Delivering my child by the planned cesarean section is convenient for me.
12. A planned cesarean section will provide relief so I can bond more with my child.
13. Delivering my child by the vaginal birth method can change my bodily form.

### Injunctive normative beliefs

14. My spouse believes that the planned cesarean section is risky for my child.
15. My spouse believes that the planned cesarean section is unsafe for me.
16. My family believes that the planned cesarean section is hazardous for my child.
17. Delivering my child by the planned cesarean section is an essential experience for my spouse.
18. To my spouse, delivering my child at a specific time of day and at a specific time of the year can impact my child’s success in life.

## Abbreviations

CFI: Comparative Fit Index
CVI: Content validity index
CFA: Confirmatory factor analysis
CVR: Content validity ratio
CDMR: Cesarean delivery on maternal request
EFA: Exploratory factor analysis
GFI: Goodness of Fit Index
ICC: Intraclass correlation coefficient
KMO: The Kaiser-Meyer-Olkin
NFI: Normed Fit Index
NNFI: Non-Normed Fit Index
RMSEA: Root Mean Square Error of Approximation
SRMR: Standardized Root Mean Square Residual
TRA: Theory of Reasoned Action
TPB: Theory of Planned Behavior
IR-TBICS: Theory-based Intention to Cesarean Section
WHO: World Health Organization
